# Repeatedly Applied Peptide Film Kills Bacteria on Dental Implants

**DOI:** 10.1007/s11837-019-03334-w

**Published:** 2019-01-18

**Authors:** CATE WISDOM, CASEY CHEN, ESRA YUCA, YAN ZHOU, CANDAN TAMERLER, MALCOLM L. SNEAD

**Affiliations:** 1. Bioengineering Program, Institute for Bioengineering Research, University of Kansas, Lawrence, USA.; 2. Herman Ostrow School of Dentistry of USC, Center for Craniofacial Molecular Biology, University of Southern California, Los Angeles, USA.; 3. Mechanical Engineering Department, University of Kansas, Lawrence, USA.; 4. Molecular Biology and Genetics Department, Yildiz Technical University, Istanbul, Turkey.

## Abstract

The rising use of titanium dental implants has increased the prevalence of peri-implant disease that shortens their useful life. A growing view of peri-implant disease suggests that plaque accumulation and microbiome dysbiogenesis trigger a host immune inflammatory response that destroys soft and hard tissues supporting the implant. The incidence of peri-implant disease is difficult to estimate, but with over 3 million implants placed in the USA alone, and the market growing by 500,000 implants/year, such extensive use demands additional interceptive approaches. We report a water-based, nonsur-gical approach to address peri-implant disease using a bifunctional peptide film, which can be applied during initial implant placement and later reapplied to existing implants to reduce bacterial growth. Bifunctional peptides are based upon a titanium binding peptide (TiBP) optimally linked by a spacer peptide to an antimicrobial peptide (AMP). We show herein that dental implant surfaces covered with a bifunctional peptide film kill bacteria. Further, using a simple protocol for cleaning implant surfaces fouled by bacteria, the surface can be effectively recoated with TiBP-AMP to regain an antimicrobial state. Fouling, cleansing, and rebinding was confirmed for up to four cycles with minimal loss of binding efficacy. After fouling, rebinding with a water-based peptide film extends control over the oral microbiome composition, providing a novel nonsurgical treatment for dental implants.

## INTRODUCTION

There is a continuing need to improve patient treatment based on the therapeutic advantages offered by titanium dental implants. However, a complex biofilm rapidly forms on the surface of any dental implant once placed in the oral cavity. Within several weeks, the biofilm consists of typical subgingival bacterial species, including keystone periodontal pathogens such as *Porphyromonas gin-givalis, Aggregatibacter actinomycetemcomitans*, *Tannerella forsythia, Treponema denticola,* and *Prevotella intermedia.*^1^ The relative abundance of commensal bacterial species to periodontal pathogens shifts with dysbiosis and too often induces the host to mount an inflammatory response leading to peri-implant diseases. This starts with peri-implant mucositis and can culminate with peri-implantitis, resulting in loss of soft and hard tissue anchoring the implant in the jaw, thus threatening and/or reducing the implant’s useful life.

The expanded use of implants in dental treatment coupled with the rising prevalence of peri-implant disease threatens to shorten implant life and lead to implant failure in increasing numbers of patients.^2,3^ Recent consensus on peri-implant disease points to plaque accumulation and microbial dysbiogenesis as the major factors triggering the host immune inflammatory response.^4–6^ Millions of implants are placed yearly around the world, with 3 million placed in the USA alone and rising at 500,000/annum.^7^ Reduced service life and eventual implant failure will therefore have a growing adverse impact on public health, with an increase in healthcare cost. On the other hand, effective treatment for peri-implant disease would make the benefits of implants available to a wider group of high-risk patients.^8,9^

Despite a high success rate, within 5 years of placement, a small percentage (4–8%) of implants fail,^10–12^ and a higher percentage fail in patients with chronic illness, advanced age, and/or poor periodontal health.^9,13–15^ A Cochrane metaanalysis report estimated the incidence of peri-implantitis as 1.6% after 3 years and 5.5% at 10 years.^16,17^ In contrast, Derks and colleagues placed the incidence of peri- implantitis at ~ 14.5% after 9 years of service,^18^ while others identified clinically significant, nonlinear bone loss as early as after 3 years of function in more than ~ 80% of patients.^18^ With implant use growing, increasing implant loss has a profound financial healthcare cost, with the potential for loss of public confidence in the dental profession.

There is currently no definitive means for controlling or eliminating the bacterial biofilm on implants.^19–21^ Current state-of-the-art treatments for implants are similar to those for periodontal disease and include mechanical debridement and/or medicinal means intended to retard the biofilm. For implant surfaces, these include abrasive cup polishing, abrasive air blasting, titanium brushes with cleansing agents such as sodium hypochlorite or povidone-iodine, chlorohexidine rinses, antibiotics, and antimicrobials. Each treatment can be used alone or in concert by dental professionals. Successful treatment for peri-implant disease must also recognize the need to maintain biocompatibility, osteogenic competency, and cell viability at the implant surface to obtain favorable host cell responses.^20–22^ Thus, a commonly held goal remains to control the oral microbiota in order to arrest or slow hard and soft tissue destruction by the host inflammatory response.

There have been numerous attempts to boost implant surface performance using biologically active signals.^23–30^ However, the majority of these have required passive absorption or chemical coupling to the surface.^31–33^ Absorption leads to leaching and poor performance. Covalent coupling has also met with limited success, because coupling agents (e.g., thiols, carboxylic acids, hydroxyl, guanidines, and amines) creates hostile environments to bioactive molecules, leading to loss of bioactivity^31,34^ and/or incorrect display of the bioactivity to the cellular environment. Moreover, these hostile coupling environments can be used only during manufacturing and not at recall appointments, long after placement, when the longevity of the implant is threatened. Ideally, both strong affinity to the implant surface and maintenance of the antimicrobial state are required.

Peri-implantitis has become a growing concern in the oral health community due to the increasing popularity of dental implants to restore form and function. Because, at present, none of the nonsur-gical treatments result in a superior outcome, there is a lack of consensus with respect to predictable treatments for peri-implantitis, making treatment choices all but unmanageable for clinicians and patients. With the number of dental implants continuing to rise, there is an urgent need to identify a strategy that can further slow or even prevent peri-implantitis. Successful approaches will require novel and rational engineering design that can leverage the multifaceted characteristics of biomolecules, as well as providing structural and functional properties at the material-tissue interface_._^35,36^

Using a combination of experimental and computational bioengineering approaches, we engineered a bifunctional peptide to provide a biocompatible, water-based, easy-to-apply, durable peptide film that exhibits antimicrobial activity. The bifunctional peptide can be repeatedly applied at recall appointments for continued treatment of peri-im-plantitis to extend implant longevity. Reapplication offers a safe, inexpensive, water-based bifunctional peptide film to treat existing implants at recall appointments, to arrest disease progression as a viable peri-implantitis treatment strategy compared with current state-of-the-art treatments.

## EXPERIMENTAL PROCEDURES

### Synthesis and Purification of Bifunctional Peptides

Peptides were produced with an AAPPTec Focus XC automated solid-phase peptide synthesizer using standard fluorenylmethyloxycarbonyl (Fmoc) chemistries and Wang resins.^23^ To remove the Fmoc protecting group, Wang resins preloaded with the first amino acid protected by a Fmoc group were treated with 20% piperidine in dimethylformamide (DMF). Fmoc deprotection was monitored by ultraviolet (UV) absorbance, and the deprotected resin was then washed with DMF to remove piperidine. Modified amino acids with protected side chains and Fmoc were solubilized in DMF at concentration of 0.2 M and added in sevenfold excess. In a separate measuring vessel, amino acids in solution were activated with 0.4 M *O*-benzotriazole-*N,N,N’,N’*-te-tramethyluronium hexafluorophosphate (HBTU) in DMF and 1 M 4-methylmorpholine (NMM). The activated amino acid was then added to the reaction vessel and mechanically mixed under nitrogen gas for 45 min to couple the amino acid to the resin. Double coupling was used for each amino acid in the sequences. After amino acid addition, the resin was washed with DMF and the protocol applied again for each subsequent amino acid.

Following synthesis, the resins with synthesized peptides were dried with reagent-grade ethanol to remove residual DMF. The peptides were cleaved from the resin, and the side-chains were deprotected using Reagent K [trifluoroacetic acid (TFA)/ thioanisole/H_2_O/phenol/ethanedithiol (87.5:5:5:2.5)] and precipitated using cold ether. Crude peptides were purified using reversed-phase (RP) high-performance liquid chromatography (HPLC), then lyophilized and stored at – 20°C.

### Minimum Inhibitory Concentration (MIC)

The MIC of TiBP-AMP was determined spectropho- tometrically against *Streptococcus mutans* bacteria. Bacteria were cultivated and grown to mid-log phase. Serial dilutions, beginning at 256 *μ*M peptide concentration, were added to wells containing a final bacterial concentration of 1 × 10^7^ colony-forming units (CFU)/mL in a 96-well plate and grown at 37°C for 24 h. Absorbance at 600 nm was monitored using a Cytation 3 microplate reader. Control samples consisted of 1 × 10^7^ CFU/mL bacteria only.

### Titanium Implant Disc Preparation

Coin-shaped titanium implant discs, 10 mm in diameter and 0.5 mm thick, made from grade 4 titanium (USC Engineering Shop) were lap polished and grit blasted with 180–220 micron titanium dioxide particles and cleaned following a published protocol.^37^ Briefly, the cleaning protocol was to sonicate the discs in water, 70% ethanol, 40% sodium hydroxide, and 50% nitric acid, followed by rinsing with water and autoclaving prior to use.

### Bacterial Culture and Maintenance

*Streptococcus mutans* ATCC 700610 was cultured according to the ATCC protocol in brain heart infusion (BHI) broth (BD Difco).^38^ Several drops of rehydrated frozen stock of bacteria were streaked on a BHI agar plate and incubated for 24 h at 37°C and 5% CO_2_. A single colony was removed from the agar plate and used to inoculate 5 mL of appropriate medium followed by incubation overnight. Bacteria were grown to mid-log phase with final concentration of 10^7^ CFU/mL.

### Bifunctional Peptide Binding on Titanium Discs

New, sterile titanium discs were functionalized with bifunctional peptides by incubation with peptide solution at 37°C, specifically 100 *μ*L of 100 *μ*M concentration of bifunctional peptide for TiBP-AMP and C-AMP. Following incubation, the discs were washed in a 24-well plate by pipetting 400 *μ*L deionized (DI) water onto the wall of a well containing the disc and then removing the H_2_O. Each disc was washed three times. The same procedure was repeated for the rebinding step for disc fouled/cleaned of bacteria.

### Fluorescence Imaging for Peptide Binding

Peptide binding was evaluated by pipetting 20 *μ*L Pierce fluorometric peptide labeling reagent directly onto the previously peptide film-coated disc surface and incubating at room temperature, protected from light for 5 min. The discs were washed by holding the disc with forceps and pipetting water at a 45° angle, allowing the water to flow across the disc face. The discs were transferred to glass microscope slides, inverted, and imaged using an inverted fluorescence microscope (Zeiss AxioPlus). The fluorescence images were saved both as a two-dimensional (2D) representation, and a three-dimensional (3D) representation in which the *z*-axis height corresponds to the fluorescence intensity. Peptide binding was quantified as the percentage surface coverage using ImageJ software version 1.52a.

### Peptide Film Antibacterial Function on Titanium Discs

Peptide-functionalized discs were evaluated for antibacterial efficacy against *S. mutans* using the BacLight Live/Dead assay kit to differentiate living from dead bacteria present on the disc surface with selected bifunctional peptides compared with a water control. *S. mutans* (ATCC 700610) bacteria were grown and cultivated according to ATCC protocols. *S. mutans* bacteria (400 *μ*L of 1 × 10^7^ CFU/mL) were incubated with functionalized discs for 4 h, then washed with 0.85% NaCl, according to manufacture recommendations, to remove any phosphates that could interfere with the Live/Dead stains. A solution of 30% stain [1:1 ratio of propidium iodide (PI) to SYTO9] in 70% 0.85% NaCl solution was used to stain bacteria on the disc surface. The discs were then transferred to a glass microscope slide and inverted for fluorescence microscopy. Images of five unique locations on the discs were collected at 10 × magnification.

### Implant Disc Cleaning for Rebinding

Discs were cleaned to remove bacteria, peptide, and salts using a method that is common to implant retreatment visits. An electric toothbrush with a round head slightly larger than the diameter of the disc was secured in a laboratory stand. A Petri dish filled with peri-plast wax was used as a base for insertion of 30-gauge needles in a triangular configuration surrounding the disc to prevent motion of the disc when the electric toothbrush was applied with 100 g force at 10 cm from the secured pivot point. A 1:10 solution of sodium hypochlorite was pipetted into the Petri dish, and the disc was brushed for 2 min, then removed and washed thoroughly with water. The disc was then air-dried in a sterile laminar flow hood in preparation for peptide rebinding. The rebinding protocol was identical to the initial binding protocol.

### Retreatment Cycle Binding and Reapplication

A solution of 33 iM TiBP-AuBP in phosphate buffered saline was pipetted onto a titanium disc and incubated at room temperature for 1 h. Discs were washed once with DI H_2_O, then dried at room temperature. A solution of 50-nm gold nanoparticles (AuNPs; Ted Pella Inc, USA) was incubated at room temperature on the peptide-functionalized disc for 20 min. The discs were washed once with DI H_2_O and dried at room temperature. Next, a solution of 8 *μ*M green fluorescent protein-gold binding peptide fusion (GFP_uv_-AuBP) was incubated on the surface for 20 min, washed, dried, and imaged using a fluorescence microscope (Zeiss AxioPlus). The same procedure was applied for four repetitions of fouling/cleansing. All images were analyzed using ImageJ software (version 1.52a).

### Surface Profilometry

Three-dimensional surface measurements of the discs were visualized using a white-light profilometer (Veeco Wyko NT 1100 optical profiler). Pictures were taken at 5 ×, 10 ×, and 50 × magnification. Nonoverlapping pictures were taken of the disc. To obtain images from the same disc coordinates, a grid was etched onto a glass slide that repeatedly oriented the disc in the same orientation and position. The profilometer was then manipulated to ensure that the same region of the disc was imaged for consistency and reproducibility.

### De Novo Peptide Structure Prediction and 3D Model Generation from Amino Acid Sequence

The de novo 3D structural modeling approach available through the online service PEP-FOLD 3.5 was implemented to generate Protein Data Bank (PDB) models for the five best models of each bifunctional peptide amino acid sequence.^4^ PEP- FOLD 3.5 generates 3D structural conformations of linear peptides of between 5 and 50 amino acids and provides PDB models for the five best structures. PEP-FOLD 3.5 generates peptide structures by assigning one of 27 structural alphabet (SA) terms to fragments of four amino acids overlapping by three. The SA generalizes the secondary structure by assigning geometric descriptors emitted by the hidden Markov model described by Maupetit et al.^5^ Three-dimensional models are then generated from the fragments using a course-grained representation and refined by 30,000 Monte Carlo steps. The chimeric peptide sequences were input into the PEP-FOLD 3.5 online service, and 200 simulations were run assuming aqueous conditions and neutral pH. Once generated, the models were clustered and sorted using the Optimized Potential for Efficient Structure Prediction (sOPEP).^7^ Nonbiased modeling was applied.

### 3D Model Visualization and Recoloring

PDB files containing the secondary structure models generated by PEP-FOLD 3.5 were visualized and further analyzed using UCSF Chimera.^8^ The structures were colored according to the peptide domains composing the bifunctional peptides. The first functional domain was colored blue, the spacer was colored black, and the second functional domain was colored red. The structures were oriented so that the first functional domain was located at the bottom. The surface was added to the ribbon structure, and transparency of 80% was applied.

## RESULTS

### Antibacterial Activity in Solution

**Fig. 1.**
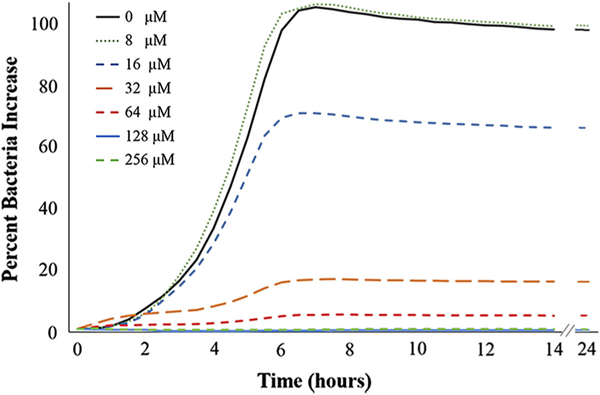
Concentration of TiBP-AMP peptide in solution required to inhibit bacterial growth of *Streptococcus mutans.* Growth was measured every 30 min for 24 h by absorbance at 600 nm and plotted as growth curves representing the increase of bacteria over time. The inhibitory concentration for TiBP-AMP was determined to be 64 *μ*M.

The bacterial growth curves of *S. mutans* bacteria grown in presence of serial dilutions of TiBP-AMP are shown in Fig. 1. The minimum concentration of TiBP-AMP required to inhibit *S. mutans* bacterial growth was found to be 64 *μ*M. The lowest concentration evaluated, viz. 8 *μ*M, showed no bacterial inhibition, while in contrast, 256 *μ*M resulted in no growth of *S*. *mutans*.

### Implant Disc Preparation and Cleansing

Discs were cleaned using a method common to periodontal therapy^39,40^ that does not adversely affect clinical attachment. Hypochlorous acid (NaOCl) and a rotary electric toothbrush (Supplementary Fig. S1) were used as a clinically relevant cleaning method to allow rebinding of bifunctional peptide film to the implant surface.

### Binding and Rebinding of the Bifunctional Peptide

**Fig. 2.**
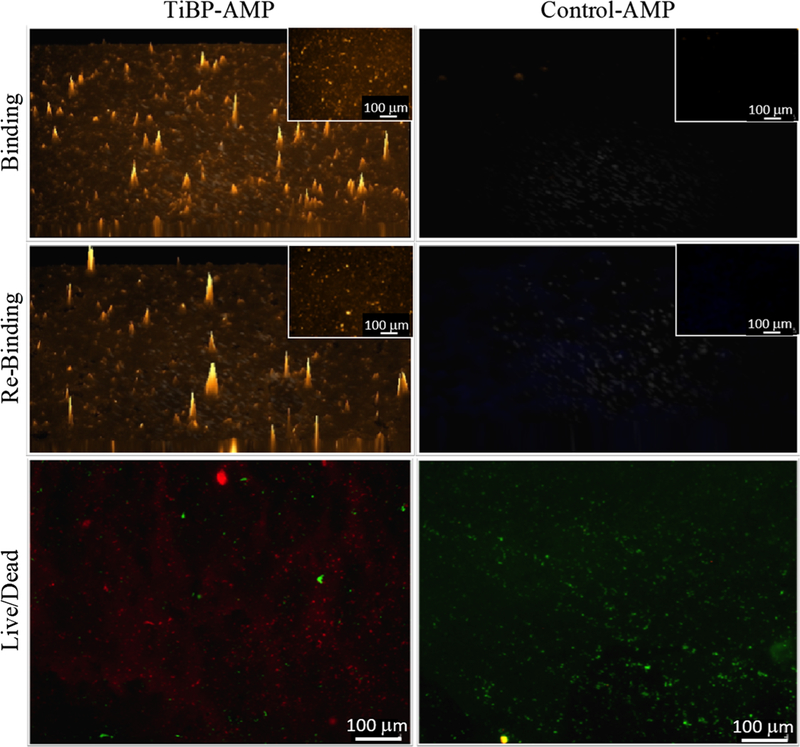
Fluorescence microscopy images of bifunctional peptide binding and antimicrobial activity on titanium implant discs. Binding and rebinding with 100 *μ*M of either bifunctional peptide was identified with a fluorescent dye. In the larger 3D images, the *z*-axis height corresponds to fluorescence intensity, while the smaller insert images correspond to 2D representations. Rebinding was performed on a once-fouled *S*.*mutans* implant disc. The antibacterial properties of the bifunctional peptides against *S.mutans* are shown using a Live/Dead (L/D) assay to differentiate live (green) from dead (red) bacteria present on the disc surface. Scale bar = 100 *μ*m.

The top panels of Fig. 2 depict TiBP-AMP and Control-AMP (AMP without the TiBP domain) binding on titanium discs. Binding was quantified as percentage surface coverage and determined to be 46.8% for TiBP-AMP and 0.2% for Control-AMP (Table I). Rebinding following bacterial fouling and a clinically relevant cleaning procedure is shown in the middle panels of Fig. 2. The cleaned disc was recoated with the same peptides, and the surface coverage for TiBP-AMP was determined to be 28.5% (Table I) compared with 0.2% for Control-AMP (Table I). After bacterial fouling, cleaned discs retained 60% of the original binding capacity. The absence of binding of the control bifunctional peptide to the cleaned discs demonstrates scant amounts of nonspecific adherence of the peptide to bacterial debris.

### Antibacterial Functionalization on Implant Discs

Discs were purposely inoculated with *S. mutans* to represent implant fouling to evaluate peptide film surface coverage on dental implant surfaces. The lower panels of Fig. 2 show the difference in the antibacterial properties between discs treated with TiBP-AMP versus Control-AMP. The percentage surface coverage of dead bacteria on the TiBP-AMP disc was 19.3%, compared with 0.2% for the control (Table II). There was a significant difference of 53.3% living bacteria on the control disc compared with 1.7% on the TiBP-AMP disc (Table II).

### Retreatment Cycle Binding and Reapplication

**Fig. 3.**
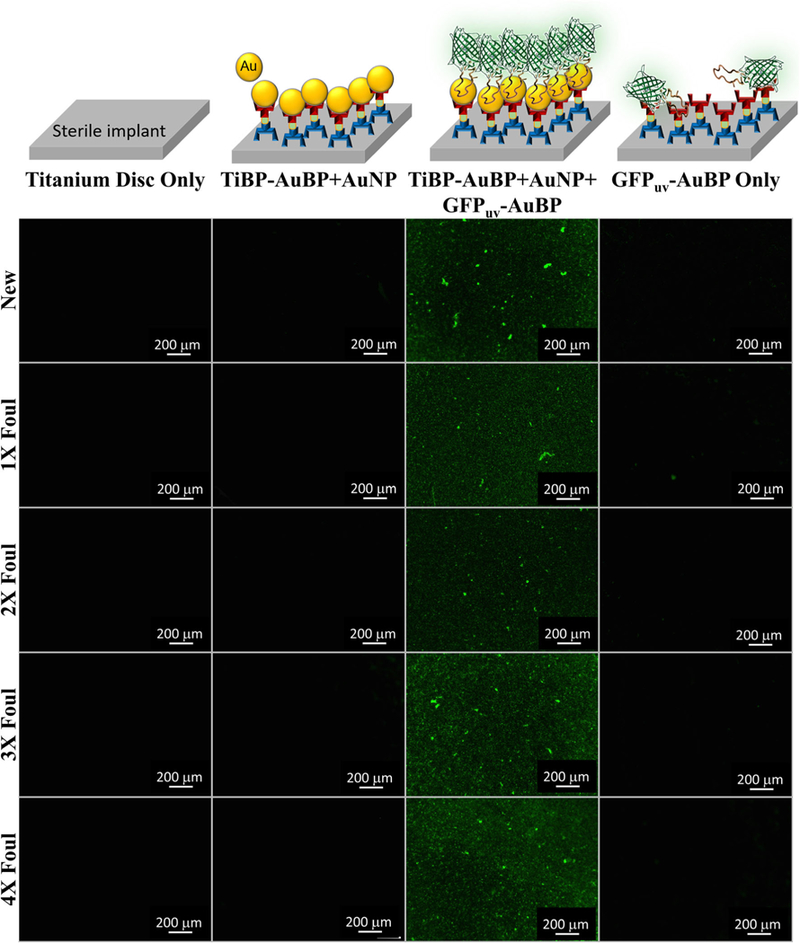
Fluorescence microscopy images of binding/rebinding on implant discs fouled multiple times by bacteria. A bifunctional peptide composed of the titanium binding peptide (TiBP) and a gold binding peptide (AuBP) was bound to the titanium disc and to gold nanoparticles (AuNP). A fusion protein, green fluorescent protein (GFP_uv_), fused to AuBP was subsequently bound to the AuNP immobilized on the titanium surface by the TiBP-AuBP bifunctional peptide. Schematics at the top of the figure represent the layer-by-layer assembly procedure used for imaging. After each addition, the surfaces were washed to remove any non-specifically bound peptides. Fluorescence images were obtained at each step and are shown. The procedure was repeated for multiple rounds of fouling to model multiple retreatment visits for implant surfaces affected by peri-implant disease. Scale bar = 200 *μ*m.

Figure 3 shows the binding on titanium discs fouled up to four times. The bifunctional peptide TiBP-AuBP was revealed by incubation with gold nanoparticles that bind to AuBP (gold binding peptide). A fusion protein with green fluorescent protein (GFP_uv_-AuBP) was used to identify peptides via the GFP fluorescence signal that were bound to the AuNPs on TiBP-AuBP film attached to the titanium disc surface in a “sandwich” technique. Minimal to no fluorescence was observed for bare titanium discs and for discs function-alized with TiBP-AuBP + AuNP, and no signal for discs functionalized with GFP_uv_-AuBP fusion protein only. The fluorescence of the discs was measured using ImageJ, and the results for TiBP-AuBP + AuNP + GFPuv-AuBP are depicted as percentage surface coverage in Table III.

### De Novo Secondary Structure Generation

**Fig. 4.**
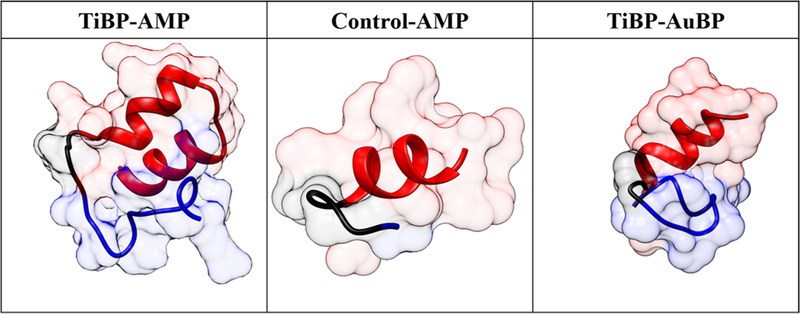
De novo secondary structures of the bifunctional peptides studied. The structures have been recolored according to the domains in each bifunctional peptide. In all structures, the titanium binding peptide is blue, the spacer peptide is black, and the antimicrobial peptide domain is red.

Secondary structures were generated for the three bifunctional peptides studied herein. The secondary structures for TiBP-AMP, Control-AMP, and TiBP-AuBP are depicted in Fig. 4. The domains were recolored, so that blue indicates the first functional domain (TiBP or Control), black indicates the spacer, and red indicates the second functional domain (AMP or AuBP).

### Titanium Disc Surface Topography

**Fig. 5.**
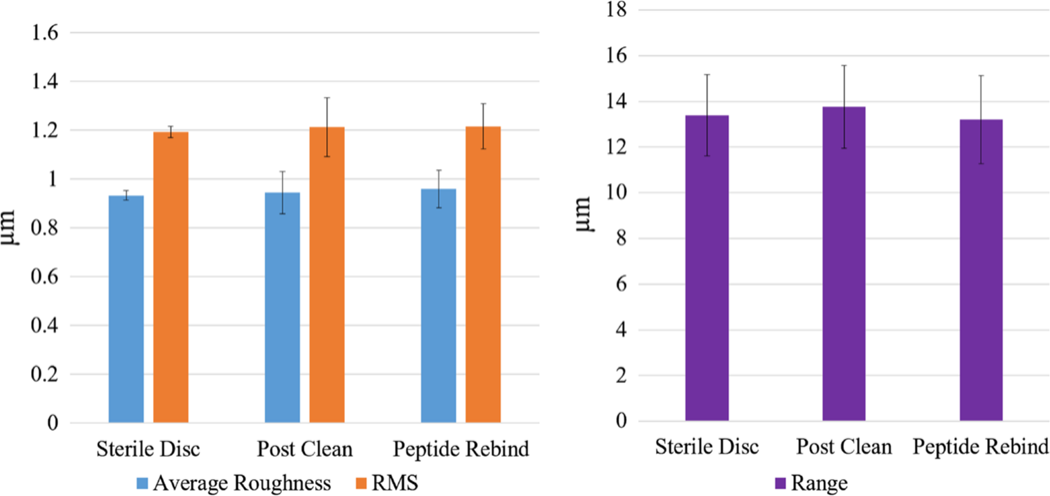
Surface topography characterization by optical profilometry. Optical profilometry images were collected and analyzed for sterile discs (Sterile), discs following bacterial fouling and the clinical cleaning procedure (Post Clean), and TiBP-AMP rebound to the fouled and cleaned disc surface. The lack of statistical difference (*n* = 5) for the average, RMS, and range of the roughness indicates that the surface topography prepared as an optimized surface for osteointegration was preserved through cleaning and rebinding

Surface topography characterization is summarized in Fig. 5. The average, root-mean-square (RMS), and range of the roughness are reported for bacteria-fouled titanium discs after cleaning and for the TiBP-AMP after rebinding to the titanium discs. There was no significant change in the surface topography characteristics among the samples. The standard deviation for the sterile discs is due to it being lap polished and then blasted with 80 mesh titanium oxide to reproduce the roughened surface which improves osteointegration. The “sterile disc” was never coated by functional film and therefore represents the air-jet-blasted rough surface.

## DISCUSSION

### Antibacterial Activity in Solution

The antibacterial activity of TiBP-AMP was established. Critical to the success of the bifunctional peptide on the surface is the antimicrobial function of the AMP domain when joined to the anchoring domain created by a titanium binding peptide (TiBP) through an engineered spacer (Fig. 4). The titanium binding peptide has been previously reported to effectively serve as a self-assembling anchor for bifunctional peptides.^23,25,41^ Similarly, we have previously established the importance of engineering the design of the spacer to optimize the function of the antimicrobial peptide domain in the bifunctional peptide construct.^41^ The spacer serves as a link between the two functional domains and is designed to preserve the secondary structure of each individual domain, a parameter that is tightly linked to antimicrobial function. Here, a novel AMP domain obtained from literature was linked to the TiBP through a “spacer5” to create a bifunctional peptide, as previously studied.^41^ The antimicrobial peptide (AMP domain) and the bifunctional peptide was selected through a rational design process based on previously determined antimicrobial “rules”.^23^ The minimum inhibitory concentration for the TiBP-spacer5-AMP bifunctional peptide was established at 64 iM for an ATCC line of *S. mutans* (Fig. 1). The bifunctional peptide can be evaluated against other oral pathogens which significantly influence the initiation and progression of peri-implant disease. Other keystone periodontal pathogens that could be evaluated include *P. gingivalis, A. actinomycetemcomitans*, *T. forsythia, T. denticola,* and *P. intermedia.*^1^ As more data are obtained on the function of this peptide construct against oral pathogens, the same “rule” induction method could be applied to elucidate peptide secondary structure features that are predominant in peptides effective against bacteria in addition to *S. mutans*.^23,42^

### Binding and Rebinding of the Bifunctional Peptide

TiBP-AMP is designed to incorporate a high-affinity titanium implant anchoring peptide with an antimicrobial peptide linked through a spacer designed to preserve the function of each peptide. The data demonstrate that the selected spacer design preserves not only the antimicrobial activity but also the robust anchoring activity to the implant surface through the TiBP domain, as seen during repeated retreatment of cleansed bacteria-fouled surfaces as would occur for implants already placed in the jaws (Fig. 2, Table I).

TiBP-AMP can be repeatedly applied to a bacte-ria-fouled implant surface to deliver antimicrobial properties to address the etiopathogenesis of peri-implant disease. In contrast, the Control-AMP (without TiBP) showed minimal absorption to the surface of bacteria-fouled and cleansed titanium discs (Fig. 2, Table I). This finding of low binding to fouled/cleaned titanium surfaces indicates that peptide binding is not due simply to nonspecific adherence/absorption to the implant surface, nor to any bacteria debris (biopolymers) retained on the fouled surface. The experimental design is based on application of known maintenance procedures followed at professional recall appointments^39,40^ demonstrate that the described bifunctional peptide can be successfully reapplied and anchored to the surface of a previously fouled and cleaned implant surface.

### Antibacterial Functionalization on Implant Discs

Antibacterial and titanium anchoring functions were demonstrated simultaneously on discs functionalized with TiBP-AMP when challenged with *S. mutans* bacteria. The TiBP-AMP film successfully demonstrated an antibacterial function with 19.3% dead bacteria, compared with 0.2% for the Control- AMP. Additionally, the TiBP-AMP peptide film had antibiofouling properties, with 21% total surface coverage by bacteria (living and dead), compared with 53.5% (Table II) for the disc treated with the control peptide. Bifunctional peptides have previously been used as antibacterial surface agents; however, we demonstrate herein their antibacterial and antibiofouling activity following rebinding using a procedure that could be performed clinically during professional retreatment appointments. This technology represents an advancement in the field over passively absorbed molecules, which are subject to leaching and poor performance challenges.^23–3^ The use of a water delivery system for the bifunctional peptide completely eliminates the need for the biologically hostile coupling environments typically required for chemically coupling bioactive molecules.^31–3^ The capacity to deliver a water-based bifunctional peptide to implants that have been previously placed in the jaw is a paradigm shift, allowing potentially limitless retreatment opportunities to control oral biofilms during the life of the implant.

### Retreatment Cycle Binding and Reapplication

The novel method applied to reveal peptide anchored on the implant surface is demonstrated in Fig. 3. This method ensures specific identification of the bifunctional peptide of interest on a titanium surface prepared for integration with bone. This technique also ensures that the bifunctional peptide, TiBP-AuBP alone was identified, avoiding noise and not due to nonspecific interactions with bacterial biopolymers on the disc surface. The inclusion of the AuBP amino acid sequence within green fluorescent protein reveals no discernible effect on gold binding.^43^ The specificity of detection was preserved following up to four cycles of fouling/cleansing. The quantitation of the peptides bound to the discs demonstrates that precise binding to the titanium surface is preserved. A *t* test performed on our generated data revealed statistical significance based on a (two-tailed) *P*-value of 0.011. Furthermore, the data demonstrate that the amount of peptide bound generally increased slightly with each successive fouling cycle, with the exception of cycle 2. The demonstrated preservation of the TiBP anchored to the surface strengthens the impact of this technology as a nonsurgical, water-based retreatment option for peri-implant disease. Multiple retreatment procedures can be performed, extending the lifetime of the implant.

### De Novo Secondary Structure Generation

The secondary structures generated demonstrate preservation of the a-helix character of the antimicrobial domain, which has previously been linked to antimicrobial function. The engineered spacer is critical to preserving the function of the antimicrobial and binding domains.

### Titanium Disc Surface Topography

Titanium implant discs were prepared by grit blasting with 180–220 micron titanium dioxide particles^37,44–46^ and sterilization following a widely used protocol similar to that used by implant manufacturers to create “active osteoin-tegration” surfaces^46^ Biocompatibility is an essential feature for all implant surfaces, and preserving the surface properties is a mandatory design requirement. The average, RMS, and range of the roughness did not change significantly after fouling and cleaning for retreatment, or after rebinding of the bifunctional peptide film (Fig. 5). These results support the advantage of this approach to create not only an antibacterial and antibiofouling implant surface, but also one that is competent to support osteointegration and is similar to standards for surface topography currently favored by implant manufacturers.

## CONCLUSION

A water-based, nonsurgical approach to address peri-implant disease using application of a bifunctional peptide film during initial implant placement of new implants or as retreatment for existing implants was demonstrated. The functions of the TiBP and AMP domains linked by an engineered spacer in a bifunctional peptide were confirmed by identification of the minimal inhibitory concentration in solution, as well as by surface antimicrobial activity. Moreover, this simple cleansing protocol likely preserved the biocompatibility of the titanium implant while reexposing the titanium surface after bacterial fouling to permit sufficient rebinding of the bifunctional peptide. The rebound bifunctional TiBP-AMP peptide film exhibited antibacterial and antibiofouling activity over four fouling/cleaning cycles. The modularity of this approach enables it to be combined with our next generation peptide engineering methodologies to improve both the titanium binding and antimicrobial properties of the peptide film, which could lead to better coverage, with higher rebinding affinity and antimicrobial effectiveness, respectively. This technology represents a paradigm shift in prevention of dental implant failure while adding to the range of bioactive molecules that can be anchored to implant surfaces to improve their function, such as osteointegration.^47^

## Supplementary Material

Supplemental

## Figures and Tables

**Table I.** Quantitative results for the binding and rebinding of the bifunctional peptides shown in Fig. 2

	TiBP-AMP (%)	Control-AMP (%)
Bind	46.8	0.2
Rebind	28.5	0.2

Results represent the percentage surface coverage of the peptide measured by ImageJ software analysis. Approximately 60% of the initial binding to a sterile implant disc was preserved on rebinding to a one-time *S. mutans*-fouled implant disc.

**Table II.** Quantitative results from the live/dead images of *S. mutans* bacteria on implant discs treated with bifunctional peptides shown in Fig. 2

	TiBP-AMP (%)	Control-AMP (%)
Live	1.7	53.3
Dead	19.3	0.2
Total coverage	21.0	53.5

Results represent the percentage surface coverage of live and dead bacteria measured by ImageJ software analysis. TiBP-AMP resulted in a more effective antibacterial and anti-biofouling surface, represented by a higher percentage coverage of dead bacteria and lower total percentage of bacterial coverage on the surface.

**Table III.** Fluorescence microscopy image quantification by measuring the percentage surface coverage of the images using ImageJ software

Treatment cycle	Surface coverage (%)
	
Sterile	18.3
1 × foul	24.3
2 × foul	8.6
3 × foul	32.9
4 × foul	41.5

The percent surface coverage of the TiBP-AuBP + AuNP + GFPuv-AuBP assembly on titanium discs is depicted for sterile new discs and for discs fouled and then cleaned for up to four retreatment cycles.

## References

[1] Listgarten MA, Periodontology 2000, 5 (1994). 10.1111/j.1600-0757.1994.tb00018.x 9673162

[2] Tarnow DP, J. Dent. Res. 95, 1 (2016). 10.1177/0022034515616557. 26701918

[3] Valente NA and Andreana S, J. Periodontal. Implant. Sci. 46, 3 (2016). 10.5051/jpis.2016.46.3.136. PMC4928203 27382503

[4] Berglundh T, Armitage G, Araujo MG, Avila-Ortiz G, Blanco J, Camargo PM, Chen S, Cochran D, Derks J, Figuero E, Hammerle CHF, Heitz-Mayfield LJA, Huynh-Ba G, Iacono V, Koo KT, Lambert F, McCauley L, Quirynen M, Renvert S, Salvi GE, Schwarz F, Tarnow D, Tomasi C, Wang HL, and Zitzmann N, J. Periodontol. 89, S313 (2018). 10.1002/jper.17-0739. 29926955

[5] Caton JG, Armitage G, Berglundh T, Chapple ILC, Jepsen S, Kornman KS, Mealey BL, Papapanou PN, Sanz M, and Tonetti MS, J. Periodontol. 89, S1 (2018). 10.1002/jper.18-0157. 29926946

[6] Salvi GE, Cosgarea R, and Sculean A, J. Dent. Res. 96, 1 (2017). 10.1177/0022034516667484. 27680028

[7] Achermann G, How will dentistry look in 2020?, Vision 2020: Simply doing more for dental professionals (2012), http://www.straumann.com.

[8] Karoussis IK, Kotsovilis S, and Fourmousis I, Clin. Oral Implants Res. 18, 6 (2007). https://doi.org/10.1111Zj.1600-0501.2007.01406.x. 10.1111/j.1600-0501.2007.01406.x 17868376

[9] Kotsovilis S, Karoussis IK, and Fourmousis I, Clin. Oral Implants Res. 17, 5 (2006). 10.1111/j.1600-0501.2005.01245.x. 16958701

[10] Muddugangadhar BC, Amarnath GS, Sonika R, Chheda PS, and Garg A, J. Int. Oral Health 7, 9 (2015). PMC4589703 26435609

[11] Moraschini V, Poubel LA, Ferreira VF, and Barboza Edos S., Int. J. Oral Maxillofac. Surg. 44, 3 (2015). 10.1016/j.ijom.2014.10.023. 25467739

[12] Derks J, Hakansson J, Wennstrom JL, Tomasi C, Larsson M, and Berglundh T, J. Dent. Res. 94, 44 (2015). 10.1177/0022034514563077. 25503901 PMC4541089

[13] Bryant SR and Zarb GA, J. Can. Dent. Assoc. 68, 2 (2002). 11869499

[14] Koldsland OC, Scheie AA, and Aass AM, J. Periodontol. 81, 2 (2010). 10.1902/jop.2009.090269. 20151801

[15] Greenstein G, Cavallaro J Jr, and Tarnow D, Dent. Clin. North Am. 54, 1 (2010). 10.1016/j.cden.2009.08.008. 20103475

[16] Esposito M, Grusovin MG, Tzanetea E, Piattelli A, and Worthington HV, Cochrane Database Syst. Rev., 6(2010) 10.1002/14651858.cd004970.pub4. 20556759

[17] Esposito M, Ardebili Y, and Worthington HV, Cochrane Database Syst. Rev., 7(2014) 10.1002/14651858.cd003815.pub4. 25048469

[18] Derks J, Schaller D, Hakansson J, Wennstrom JL, Tomasi C, and Berglundh T, J. Dent. Res. 95, 1 (2016). 10.1177/0022034515608832. 26701919

[19] Lang NP, Wilson TG, and Corbet EF, Clin. Oral Implants Res., 11 Suppl 1(2000). 10.1034/j.1600-0501.2000.011s1146.x 11168263

[20] Esposito M, Grusovin MG, and Worthington HV, Cochrane Database Syst. Rev., 1(2012) 10.1002/14651858.cd004970.pub5. PMC6786958 22258958

[21] Grusovin MG, Coulthard P, Worthington HV, George P, and Esposito M, Cochrane Database Syst. Rev., 8(2010) 10.1002/14651858.cd003069.pub4. PMC6866073 20687072

[22] Louropoulou A, Slot DE, and Van der Weijden F, Clin. Oral Implants Res. 26, 7 (2015). 10.1111/clr.12365. 24641774

[23] Yucesoy DT, Hnilova M, Boone K, Arnold PM, Snead ML, and Tamerler C, JOM 67, 4 (2015). 10.1007/s11837-015-1350-7. PMC4450091 26041967

[24] Gitelman A and Rapaport H, Langmuir 30, 16 (2014). 10.1021/la500310n. 24694202

[25] Yazici H, Fong H, Wilson B, Oren EE, Amos FA, Zhang H, Evans JS, Snead ML, Sarikaya M, and Tamerler C, Acta Biomater. 9, 2 (2013). 10.1016/j.actbio.2012.11.004. PMC4410049 23159566

[26] Vidal G, Blanchi T, Mieszawska AJ, Calabrese R, Rossi C, Vigneron P, Duval JL, Kaplan DL, and Egles C, Acta Biomater. 9, 1 (2013). https://doi.org/10.1016Zj.actbio.2012.09.003. 10.1016/j.actbio.2012.09.003 PMC3508072 22975628

[27] Beyeler M, Schild C, Lutz R, Chiquet M, and Trueb B, Exp. Cell Res. 316, 7 (2010). 10.1016/j.yexcr.2009.12.019. 20043904

[28] Reyes CD, Petrie TA, Burns KL, Schwartz Z, and Garcia AJ, Biomaterials 28, 21 (2007). 10.1016/j.biomaterials.2007.04.003. PMC2034748 17448533

[29] Auernheimer J, Zukowski D, Dahmen C, Kantlehner M, Enderle A, Goodman SL, and Kessler H, ChemBioChem 6, 11 (2005). 10.1002/cbic.200500031. 16206226

[30] Schliephake H, Scharnweber D, Dard M, Rossler S, Sewing A, Meyer J, and Hoogestraat D, Clin. Oral Implants Res. 13, 3 (2002). 10.1034/j.1600-0501.2002.130312.x. 12010163

[31] Castner DG and Ratner BD, Surf. Sci. 500, 1–3 (2002). 10.1016/s0039-6028(01)01587-4.

[32] Tomsia AP, Launey ME, Lee JS, Mankani MH, Wegst UGK, and Saiz E, Int J. Oral. Maxillofac. Implants, 26(2011). PMC3087979 21464998

[33] Cranford SW, de Boer J, van Blitterswijk C, and Buehler MJ, Adv. Mater. 25, 6 (2013). 10.1002/adma.201202553. 23297023

[34] Maia FR, Bidarra SJ, Granja PL, and Barrias CC, Acta Biomater. 9, 11 (2013). 10.1016/j.actbio.2013.08.004. 23933486

[35] Koidou VP, Argyris PP, Skoe EP, Mota Siqueira J., Chen X, Zhang L, Hinrichs JE, Costalonga M, and Aparicio C, Biomater. Sci, 6, 7(2018) 10.1039/c8bm00300a. PMC6019193 29850754

[36] Wohlfahrt JC, Evensen BJ, Zeza B, Jansson H, Pilloni A, Roos-Jansaker AM, Di Tanna GL, Aass AM, Klepp M, and Koldsland OC, Int. J. Implant Dent. 3, 1 (2017). 10.1186/s40729-017-0098-y. 28776288 PMC5543013

[37] Ronold HJ, Lyngstadaas SP, and Ellingsen JE, Biomaterials 24, 25 (2003). 10.1016/s0142-9612(03)00256-4 12950998

[38] ATCC, ATCC Bacterial Culture Guide, https://www.atcc.org/~/media/PDFs/Culture%20Guides/ATCC_Bacterial_Culture_Guide.ashx.

[39] Bizzarro S, Van der Velden U, and Loos BG, J. Clin. Periodontol. 43, 9 (2016). 10.1111/jcpe.12578. 27169789

[40] Jurczyk K, Nietzsche S, Ender C, Sculean A, and Eick S, Clin. Oral Investig. 20, 8 (2016). 10.1007/s00784-016-1711-9. 26759339

[41] Wisdom C, VanOosten SK, Boone KW, Khvostenko D, Arnold PM, Snead ML, and Tamerler C, J. Mol. Eng. Mater. 4, 01 (2016). 10.1142/S2251237316400050 PMC5604879 28936427

[42] Boone K, Camarda K, Spencer P, and Tamerler C, BMC Bioinformatics, 19(2018). 10.1186/s12859-018-2514-6. PMC6282327 30522443

[43] Yuca E and Tamerler C, JOM (in press). 10.1007/s11837-018-03325-3. PMC8211090 34149269

[44] Ronold HJ, Lyngstadaas SP, and Ellingsen JE, J. Biomed. Mater. Res. A 67, 2 (2003). 10.1002/jbm.a.10580. 14566794

[45] Ronold HJ, Ellingsen JE, and Lyngstadaas SP, J. Mater. Sci. Mater. Med. 14, 10 (2003). 10.1023/a:1025622407727 15348520

[46] Monjo M, Ramis JM, Ronold HJ, Taxt-Lamolle SF, Ellingsen JE, and Lyngstadaas SP, Clin. Oral Implants Res. 24, 9 (2013). 10.1111/j.1600-0501.2012.02496.x. 22587025

[47] Zhou Y, Snead ML and Tamerler C, Nanomedicine. 11, 2 (2015). 10.1016/j.nano.2014.10.003. PMC4330108 25461292

