# Supplementary material for: Repeatedly Applied Peptide Film Kills Bacteria on Dental Implants
Source: JOM (1989). Author manuscript; Available in PMC 2019 Jun 5. (PMC6550465; doi:10.1007/s11837-019-03334-w)
Supplement: Supplemental [file NIHMS1008687-supplement-Supplemental.pdf]

Supplemental Material for JOMJ-D-18-01319

"Repeatedly applied peptide film kills bacteria on dental implant".

[1] Cate Wisdom, B.S., [2] Casey Chen, D.D.S., Ph.D., [4] Esra Yuca, Ph.D., [2] Yan Zhou, Ph.D. [1,3] Candan Tamerler, Ph.D., and [1,2,#] Malcolm L. Snead, D.D.S., PH.D.,

1. Bioengineering Program, University of Kansas
2. Herman Ostrow School of Dentistry of USC, Center for Craniofacial Molecular Biology, University of Southern California
3. Mechanical Engineering Department, University of Kansas
4. Molecular Biology and Genetics Department, Yildiz Technical University

#, authors for correspondence, mlsnead@usc.edu, ctamerler@ku.edu

Supplementary Information

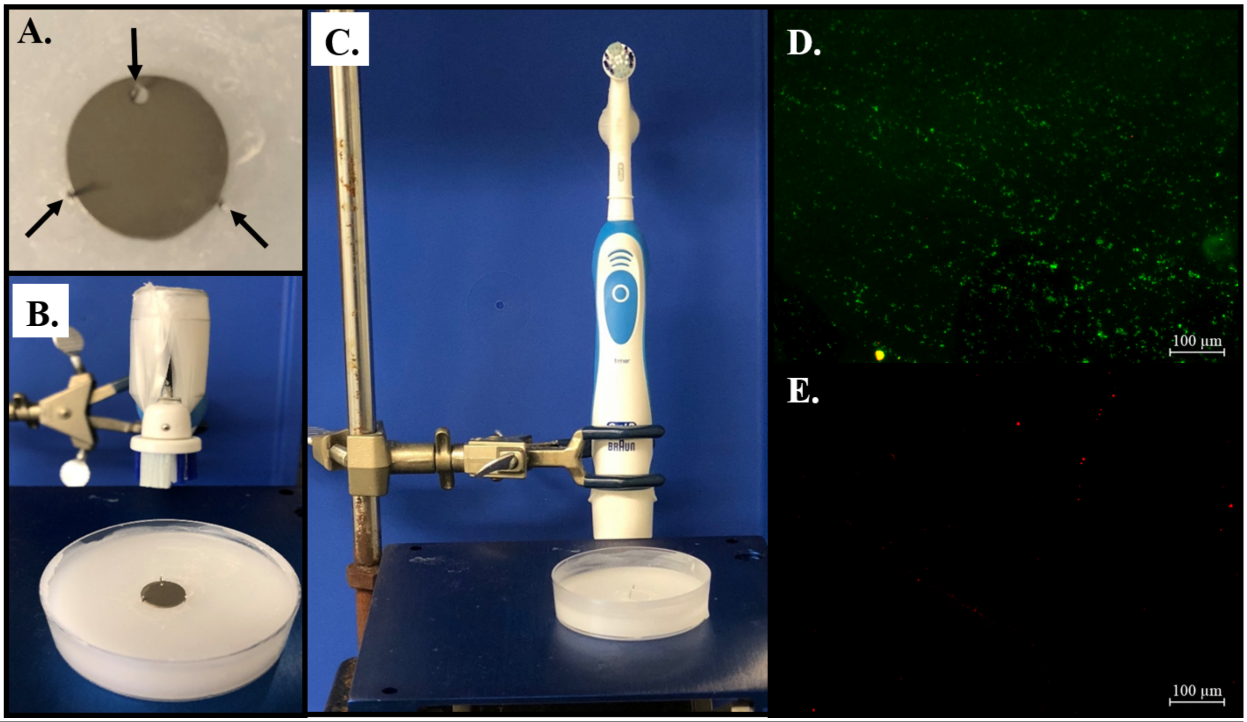

Supplementary Information 1: Cleansing of bacteria fouled titanium discs. Discs were cleaned with an Oral-B rotary electric toothbrush, using a 50g load. The fouled disc was immobilized on a paraffin-coated petri to prevent disc motion, brushed for 2 minutes with 0.6% NaOCl and washed with DI water. Bacteria on the disc surface were visualized with live/dead stain before cleansing (D) and after the cleaning procedure (E).
